# Health implications of fructose consumption: A review of recent data

**DOI:** 10.1186/1743-7075-7-82

**Published:** 2010-11-04

**Authors:** Salwa W Rizkalla

**Affiliations:** 1 INSERM, U872, équipe 7 Nutriomique, Université Pierre et Marie Curie-Paris 6, Centre de Recherche des Cordeliers, UMR S 872, Paris, 75006 France; 2 Centre de Recherche Nutrition Humaine, Ile de France, Assistance Publique-Hôpitaux de Paris, Hôpital Pitié-Salpêtrière, Département de Nutrition et d'Endocrinologie, Paris, 75013 France

## Abstract

This paper reviews evidence in the context of current research linking dietary fructose to health risk markers.

Fructose intake has recently received considerable media attention, most of which has been negative. The assertion has been that dietary fructose is less satiating and more lipogenic than other sugars. However, no fully relevant data have been presented to account for a direct link between dietary fructose intake and health risk markers such as obesity, triglyceride accumulation and insulin resistance in humans. First: a re-evaluation of published epidemiological studies concerning the consumption of dietary fructose or mainly high fructose corn syrup shows that most of such studies have been cross-sectional or based on passive inaccurate surveillance, especially in children and adolescents, and thus have not established direct causal links. Second: research evidence of the short or acute term satiating power or increasing food intake after fructose consumption as compared to that resulting from normal patterns of sugar consumption, such as sucrose, remains inconclusive. Third: the results of longer-term intervention studies depend mainly on the type of sugar used for comparison. Typically aspartame, glucose, or sucrose is used and no negative effects are found when sucrose is used as a control group.

Negative conclusions have been drawn from studies in rodents or in humans attempting to elucidate the mechanisms and biological pathways underlying fructose consumption by using unrealistically high fructose amounts.

The issue of dietary fructose and health is linked to the quantity consumed, which is the same issue for any macro- or micro nutrients. It has been considered that moderate fructose consumption of ≤50g/day or ~10% of energy has no deleterious effect on lipid and glucose control and of ≤100g/day does not influence body weight. No fully relevant data account for a direct link between moderate dietary fructose intake and health risk markers.

## Introduction

Fructose, a natural sugar found in many fruits, is consumed in significant amounts in Western diets [1]. In equal amounts, it is sweeter than glucose or sucrose and is therefore commonly used as a bulk sweetener.

An increase in high fructose corn syrup, as well as total fructose, consumption over the past 10 to 20 years has been linked to a rise in obesity and metabolic disorders [2]. This raises concerns regarding the short and long term effects of fructose in humans.

## Why is fructose of concern?

Fructose has been claimed to be of concern due to several factors: First, in the 1980's, sucrose was replaced to a large extent, particularly in North America, by high fructose corn syrup (HFCS) in carbonated beverages. The intake of soft drinks containing HFCS has risen in parallel with the epidemic of obesity [3]. Second, dietary fructose has been implicated in risk factors for cardiovascular disease (CVD): 1. Plasma triglycerides (TG) and VLDL-TG increased following the ingestion of large quantities of fructose; 2. Fructose intake has been found to predict LDL particle size in overweight schoolchildren [4]; 3. A positive relationship has been demonstrated between fructose intake and uric acid levels [5]. Third, the use of fructose as a sweetener has increased. The third National Health Examination Survey (NHANES) demonstrated that over 10% of Americans' daily calories were from fructose [6]. These studies suggest that the relationship between fructose and health needs re-evaluation.

## Fructose consumption and body weight

Lipogenesis from fructose consumption may theoretically be greater than that induced after eating other types of sugars such as glucose and sucrose [7]. But could this be physiologically true?

### Evidence from experimental studies in animals

The evidence of the action of dietary fructose, but not glucose, on increasing appetite and food intake in acute-term studies has been derived mainly from experimental studies in animals. Although glucose and fructose utilize the same signaling pathway to control food intake, they act in an inverse manner and have reciprocal effects on the level of the hypothalamic malonyl-CoA, a key intermediate in the hypothalamic signal cascade that regulates energy balance in animals [8]. When injected into the cerebroventricles of rats, fructose has been found to induce increase in food intake via a reduction of hypothalamic malonyl-CoA levels, whereas similar concentrations of injected glucose increased malonyl- CoA suppressing appetite-agonist and food intake [9]. The rapid initial steps of central fructose metabolism deplete hypothalamic ATP level, whereas the slower regulated steps of glucose metabolism elevate hypothalamic ATP level. Consistent with its effects on the [ATP]/[AMP] ratio, fructose increases phosphorylation/activation of hypothalamic AMP kinase causing phosphorylation/inactivation of acetyl-CoA carboxylase, whereas glucose mediates the inverse effects.

The question has been raised as to whether fructose may induce the same effects if presented in the systemic circulation and not injected directly in the brain. Consequently, Cha et al [10], demonstrated that when glucose was administered intra-peritoneally, hence entered the systemic circulation, it was rapidly metabolized by the brain, increasing the level of hypothalamic malonyl-Co-A. Fructose administration, however, had the opposite effect on malonyl-Co-A and food intake. Such a finding might appear to set off another alarm bell about the problems of dietary fructose. However, closer inspection reveals that the latter study used only 4 mice, which were injected with a dose of 4g/Kg of body weight, a dose too large to be considered relevant to human nutrition. While this paper demonstrated that high doses of fructose and glucose acted on different pathways, the physiological significance of these results remains unclear. Fructose ingestion is unlikely to increase fructose levels in the cerebrospinal fluid, and plasma fructose levels will never exceed the micromolar range under physiological conditions. Some authors suggested the uncertainty of these effects [11]. Therefore, no evidence of cause for health concern could be drawn from such acute studies in rodents.

The effects of fructose on body weight were further questioned. When rats were fed a high fructose diet (60%) for 6 months then switched to a high fat diet for 2 weeks, leptin levels increased and a state of leptin resistance was found prior to increased adiposity and body weight induced by the high fat diet [12]. However, in other shorter term studies (3-6 weeks) high fructose feeding (57% in weight) induced insulin resistance and hypertriglyceridemia in rats but failed to induce an increase in body weight [13-15].

Thus, in rodents while excessively high fructose intake may increase appetite by different mechanisms, its' effect on body weight needs long term dietary periods.

### Acute studies in humans: fructose, food intake and satiety

Sugars and sugar sweetened beverages have been blamed for causing obesity, but the debate has raged for many years with little resolution [16]. More recently, the intensity of the debate was fuelled by the hypothesis that HFCS lead to obesity because fructose bypasses food intake regulatory system (insulin and leptin) and favors lipogenesis [17]. It was hypothesized that energy containing drinks, especially those sweetened with HFCS promotes energy imbalance and thereby play a role in the development of obesity. In an acute-term study [17], 12 normal -weight women consumed meals containing 55, 30 or 15% of total calories as carbohydrate, fat and proteins with 30% of Kcal as either fructose sweetened or glucose sweetened beverages. As expected, glucose excursions and insulin secretion were lower after fructose meals than after glucose ones. This was associated to a decrease in leptin levels, which is an expected consequence of lowering insulin levels. It is important to notice that the reduction in leptin levels remained within physiological normal levels and fluctuated between: 9 ng/ml during the morning and 19.8 ng/ml by night. After this acute- term study, following only one meal, the authors rapidly suggested that because insulin and leptin (the main regulatory factors of food intake) were lower after fructose meals; they might increase caloric intake and ultimately contribute to weight gain and obesity. Fructose meals should be compared to sucrose the usual sugar and not to glucose which gives extreme levels.

The question was then raised whether HFCS has different effect on satiety than other isoenergetic drinks as sucrose or milk; again this question was investigated in an acute study. In order to have a simple response Soenen and Westerterp [18] compared the satiating effects of 4800 ml of HFCS, sucrose and milk containing each 1.5 MJ in comparison with a diet drink with no energy. They measured satiety by a visual analogue scale and by determining the satiety hormones (leptin and ghrelin) concentrations. They concluded that energy balance consequences were the same between the three isoenergetic drinks evaluated. Therefore, fructose in term of satiety is not different from that of usually consumed sugar and even that of another isocaloric drink (milk).

In another study Akhavan et al [19] aimed to evaluate whether HFCS in soft drinks is different from sucrose solutions. They compared solutions containing sucrose, HFCS, or various ratios of glucose to fructose (G50:F50) on food intake, average appetite and on plasma concentrations of glucose, insulin and ghrelin. Measurements were taken from base line to 80 minutes only. The authors of the latter paper concluded that all the solutions tested do not have significantly different effects on subjective and physiologic measures of satiety at a subsequent meal. Therefore, there is no solid evidence that sucrose, when consumed in its intact form, would confer any benefits over HFCS, which contains the 2 unbound monosaccharides.

Similarly, in a 24 hour study Stanhope 2008 [20] and Melanson [21] did not find substantially different effects between meals with either sucrose or HFCS on 24 hour plasma glucose, insulin, leptin and ghrelin levels. Even TG profiles were found to be similar between the two tests. These responses were found to be intermediate between the lower responses after the pure fructose syrup consumption and the higher responses after glucose solution ingestion. There was no difference in food intake during a meal consumed 50 min later or in the components of food intake regulatory mechanisms.

### Chronic studies in humans

Although acute fructose consumption could not stimulate leptin secretion, an increase in fasting leptin levels was detected after chronic high fructose intake (1 to 4 weeks) in healthy individuals, which may suggest that high fructose feeding may suppress food intake in the long term [22]. Another long term study in overweight/obese humans showed no change in body weight after 10 week-supplementation with glucose or fructose, indicating that the effect of fructose or glucose on food intake might not differ on long term bases [23].

In a cluster randomized controlled study [24], the effect of a focused educational intervention program on carbohydrate sweetened beverage consumption and overweight was studied using 644 children (7-11 years old). Children participated in a program designed to emphasize the consumption of a balanced diet and to discourage the consumption of sweetened drinks (mainly by sucrose: glucose/fructose). Sweetened drink consumption decreased in the intervention group and increased in the control one. Parallel changes in BMI occurred in each group, but without any difference between the two groups. Therefore, no conclusion could be given on appetite or body weight even if fructose is present as a part of sucrose.

### Epidemiological studies

The recent epidemiological study of Vos et al [6] created new concern in regards to fructose consumption. These authors analyzed data from the US population who had participated in the NHANES III study, collected from 1988 to 1994. 21,483 adults and children 2 years of age or older were included in this study. Investigators found that fructose consumption had increased to 54.7g/d (10.2% of total caloric intake), compared to 37 g/d (8%) of total intake in 1977-1978. The consumption was highest among adolescents (12-18 years) at 72.8g/d (12.1% of total calories). They showed that over 10% of Americans' daily calories were from fructose [6].

Bray et al [25] suggested that the increase in obesity in the last 35 years has paralleled the increasing use of high-fructose corn syrup (HFCS), which first appeared just before 1970. Current soft drinks and many other foods are sweetened with this product because it is inexpensive and has useful manufacturing properties. The fructose in HFCS and sugar makes beverages very sweet, and this sweetness may underlie the relationship between obesity and soft drink consumption. Indeed in the United States, HFCS has increasingly replaced sucrose in many foods and sweetened beverages, a fact that might appear to strengthen the hypothesis that there is a relationship between fructose and obesity. The parallelism between the increase in the consumption of high fructose corn syrup and dietary fructose and the rise in obesity over the past 10-20 years, linked fructose to the rise in obesity and metabolic disorders, mainly in the United States.

This is not the case in Europe or outside the United states, where fructose is consumed mainly from sucrose and fructose consumption is linked mainly to sugar consumption. Moreover, the evidence from metabolism studies on fructose alone is irrelevant to the HFCS and weight gain debate. Most of the studies dealing with the causes of obesity and over-weight have centered on HFCS [26].

#### Cross-sectional studies

In a cross sectional study, when correlating the BMI of the NHANES 1988-1994 cohort to the results of 24 hour dietary recall and one food frequency questionnaire by a multivariate regression model, a positive association was found between consumption of carbonated soft drinks and the BMI of females [27]. Using a continuing survey of food intake for individuals (CSFII) in another cross-sectional study, Forshee et al [28] found that BMI had a statistically positive relationship with diet carbonated soft drink consumption for both boys and girls (n= 1749) children (6-11 years) and adolescents (12-19 years). Other cross-sectional studies in American children demonstrated a positive correlation between soft drinks and BMI [29,30]. When looking at Pacific Island children living in New Zealand, where HFCS is very limited, the consumption of sucrose has been evaluated and correlated to body weight. The obese children consumed more of all types of food with no difference between obese and non obese children's consumption patterns [30].

Most of the cross-sectional studies included no controls for sedentary behaviors, physical activity, and energy intake from other sources other than beverages in the model. Moreover, in these studies BMI and beverage consumption were self-reported and hence subject to measurements errors. Causal relationship cannot be made from cross-sectional study design.

**In longitudinal epidemiologic studies, **such as the US Growing Up Today Study (GUTS) in a cohort of more than 10,000 males and females (9-14 years in 1996), authors did not find a correlation between BMI and snack food consumption, including sugar-sweetened beverages [31] when controlling for total energy [32]. In the North Dakota Special Supplemental Nutrition Program for Women, Infants, and Children (WIC) [33], no significant association was detected between any of the beverages evaluated and BMI. Even in another study among 30 children aged 6-13 years attending the Cornell Summer Day in 1997 [34], excessive sweetened drink consumption (>370g/day) did not correlate with weight gain. Again results of these longitudinal studies are not conclusive. Most of the positive correlations presented disappeared when corrected by total energy.

**Meta-analysis **linking soft drink consumption and body weight demonstrated conflicting results. One meta-analysis of 12 studies in children and adolescents [35] failed to find a positive association between soft drink consumption and body weight, where as another meta-analysis dealing with 88 studies found an association [36].

### Conclusion

The relation between HCFS and obesity has been derived mainly from epidemiological studies trying to relate the increase in consumption of dietary fructose and HFCS on one hand and to the increase in obesity (see ref [37]. In the epidemiological, cross -sectional and longitudinal studies, the overall evidence for a positive correlation between consumption of soft drinks and overweight is limited. Causal inferences cannot be made from cross-sectional study designs with values subjected to measurement error. The interventional acute studies (24 hours) found that fructose is thought to be associated with insufficient secretion of insulin and leptin and suppression of ghrelin when compared with pure glucose. Such a difference, however, could not be demonstrated when HFCS compared with sucrose, the commonly consumed sweetener. In addition appetite and energy intake do not differ in the acute-term. There are no long-term interventional studies investigating the direct relationship between HFCS and body weight [38], with the exception of Tordoff et al [39] who compared the consumption of 4 bottles of soda/day (1135g) as HFCS or as soda sweetened with aspartame for 3 weeks. Unsurprisingly, subjects who consumed the HFCS as extra calories gained more weight than those consuming the soda with aspartame. There is evidence that body weight increases when calorie intake is in a positive balance, regardless of whether this is due to HCFS, fat, proteins or any other form of calories. Moreover, in a recent meta-analysis, no significant effect of fructose consumption could be demonstrated on body weight with doses ≤ 100g/day in adults [40]. Unfortunately the recent focus on HFCS has done little to resolve the role of sugars in contributing to energy imbalance.

Meanwhile, a positive effect of fructose on satiety was demonstrated in the 1990's. The group of Rodin et al [41-43] demonstrated that the intake of 50 g fructose alone as the sole source of carbohydrate, either in solution or in the form of puddings 2 hours 25 minutes before a meal, caused a decrease in appetite and lipid intake. Therefore, this could even be used as an adjunct to weight control efforts.

#### Important points

It is clear that fructose is poorly absorbed from the digestive tract when it is consumed alone. However, absorption improves when fructose is consumed in combination with glucose and amino acids [44]. In addition, the principal sweetener in soft drinks in the US, HFCS, is not pure fructose but a mixture of fructose (55%) and glucose (45%). HFCS is predominately present as HFCS-55 (55% fructose, 41% glucose, and 4% glucose polymers) or HFCS-42 (42% fructose, 53% glucose and 5% glucose polymers) [26]. Therefore, the term "high fructose corn syrup" is not a good descriptor of its composition, but the term was mandated to distinguish the newly developed fructose-containing corn syrup from traditional all-glucose corn syrups. Factors that may account for the different effects of fructose alone or a mixture of fructose and glucose could be its gastrointestinal effects and absorption characteristics [45].

It should also be noted that even in a study that increased further the concerns about fructose intake [4], which looked at overweight Swiss children, the authors could not demonstrate any correlation between fructose intakes and adiposity or any other lipid variables in children (cholesterol, triglycerides), with the exception of LDL particle's size.

Clearly fructose itself is not driving the obesity epidemic, but there is evidence supporting the possibility that refined carbohydrates in general could have a contributory role, if not a major one. Very recently, this problem has been attributed to all added sugars (high- fructose corn syrup or fruit-juice concentrates), and not only added fructose [46].

Fructose intake as well as HFCS may be a contributor, but it's not the sole problem. Obese subjects consume too many calories for their activity level, including too much fat, protein and sugar. It is clear that energy imbalance for most individuals is caused by energy intake exceeding expenditure. A dietary solution to obesity remains elusive, but focusing on reducing one food item is unlikely to succeed[47,48]. Moreover, overweight and obesity are influenced by many genetic [49-51] and environmental factors [52]: for instance:

a) promoting water consumption can prevent overweight among children in elementary school [53]; b) habituation on behavioral and physiological responses to repeated presentations of food [54]; c) addressing specific eating patterns [55] and d) efforts to reduce fast food portion size [56].

Whatever the cause of obesity, based on the currently available evidence, an expert panel formed by the Centre of Food Nutrition and Agriculture Policy concluded that HFCS does not appear to contribute to overweight and obesity any differently than other energy sources [26].

## Fructose, lipogenesis and cardiovascular risk factors

Another concern with fructose intake is that it may induce hypertriglyceridemia and lipogenesis. **Theoretically**, fructose consumption can result in increasing TG synthesis [57].

### Intestinal absorption

fructose is absorbed from the intestine via glucose transporters 5 (GLUT 5), then it diffuses into the blood vessels through GLUT 2 or 5 [58], but mainly by GLUT 2. Contrary to glucose, fructose absorption from the intestinal lumen does not require ATP hydrolysis and is independent of sodium absorption, which results in massive fructose uptake by the liver [59].

### Hepatic metabolism (Figure 1)

**Figure 1:**
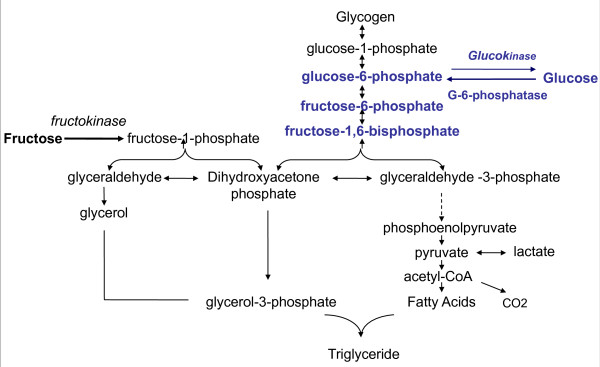
**Fructose and glucose metabolism in liver cells:** After several steps glucose is converted into fructose1,6-bi-phosphate. A reaction regulated by the rate-limiting enzyme phosphofructokinase, which is inhibited by ATP and citrate. Altogether the conversion of glucose to pyruvate is regulated by insulin. On the other hand, **fructose**, is massively taken by the liver, and converted rapidly to triose-phosphate independently of insulin control and without a feedback by ATP or citrate. A large portion of fructose is converted into glucose which can be released in the blood or stored as glycogen. A part is converted into lactate. A small portion is converted into fatty acids, which may play an important role in the development of hypertriglyceridemia and fatty liver.

The hepatic metabolism of fructose differs also greatly from that of glucose. Contrary, to glucose, fructose is metabolised exclusively in the liver by fructokinase (Km: 0.5 mM). Glucose, however, tends to be transported to the liver but could be metabolized anywhere in the body by glucokinase (Km of hepatic glucokinase: 10mM).

In the liver glucose is first phosphorylated by glucokinase to give glucose-6-phosphate, which is then converted to fructose -6-phosphate, and further to fructose 1,6-bisphosphate. This process is regulated by the rate-limiting enzyme phosphofructokinase, which in inhibited by ATP and citrate. Fructose 1,6-bisphosphate is converted into pyruvate prior to entry into the Krebs cycle. The hepatic conversion of glucose to pyruvate is regulated by insulin.

In contrast, the conversion of fructose into triose-Phosphate is a rapid process independent of insulin. Fructose bypasses the main regulatory step of glycolysis (the conversion of glucose-6-phosphate to fructose 1,6-bisphosphate controlled by phosphofructokinase) and hence can continuously enter the glycolytic pathway. This rapidity is due mainly to the low Km of fructokinase for fructose, and the absence of negative feedback by ATP or citrate [60]. A portion of triose-phosphate produced from fructose can subsequently be converted into pyruvate and oxidized into CO2 and water. Another portion is converted into lactate to be released into the circulation [61]. The major portion of the triose-phosphate produced from fructose is converted into glucose and glycogen through gluconeogenesis [62]. At the end, part of the carbons from fructose can be converted to fatty acids. Simultaneously, fructose inhibits hepatic lipid oxidation favouring fatty acid reesterification and VLDL-triglyceride synthesis [63]. Therefore, fructose can rapidly and without any control produce glucose, glycogen, lactate, and pyruvate, providing both the glycerol and acyl portion of acyl-glycerol molecules. These particular substrates and the lack of regulation of this pathway could result in large amounts of TG that can be packed into very-low density lipoproteins by the liver.

It is essential to note that the general disposition of fructose carbon between its major end products is modified by nutritional and endocrine status [64]. Once fructose has been catabolized to three-carbon molecules its subsequent metabolic fate is identical to that of glucose. Hence, fructose can also be converted to glycogen once a positive energy balance has been established. On the other hand, glucose is mainly stored as glycogen in the liver, but high glucose levels may increase formation of glycerol -3 phosphate and accelerate hepatic triglyceride production [65].

### TG Clearance

Moreover, as VLDL goes into the bloodstream, these TG can be hydrolyzed by lipoprotein lipase (LPL) into non-estrified fatty acids and monoacylglycerol. These components could be taken by adipose tissue to re-synthesise TG. However, fructose consumption does not lead to insulin stimulation resulting then in low insulin excursions that may affect LPL-stimulated lipolysis and thus contribute to reduced TG clearance. Therefore, fructose consumption has been suggested to induce both increased hepatic TG that can be packed into very-low density lipoproteins by the liver and reduced TG clearance by adipose tissue.

### Intestinal origin of TG

Fructose-induced hyperlipidemia has been also hypothesized to be of intestinal origin. Jeppesen et al [66] demonstrated that the addition of 50 g fructose to an oral fat load (40 g) resulted in higher postprandial concentrations of triglycerides and retinyl palmitate in plasma and lipoprotein fraction (of intestinal origin). These results were found to be more pronounced with high fasting plasma triglyceride concentrations. The increase in plasma TG induced by high fructose diet in hamsters, was demonstrated to originate from fructose conversion into fatty acids within the enterocytes, with overproduction of apoB-48-containing lipoprotein [67,68].

### Evidence from experimental studies in animals

Evidence of fructose induced lipogenesis comes mainly from studies in rodents [69,70]. In fact, evidence exists that consuming large amounts of fructose leads to the development of a complete metabolic syndrome in rodents [71-73].

*In the liver*, the ability to metabolize fructose more rapidly than glucose into different metabolites has been demonstrated in rats [74]. The ratio of fructose metabolism/glucose metabolism (F/G) varies between a minimum of 3 for lactic acid, pyruvic acid, CO2 and free fatty acids, and a maximum of 19 for glyceraldehyde-glycerol.

On the other hand, it has been demonstrated that feeding rats with 75% (w/w) fructose or glucose diets increased the capacity for triglyceride formation from glycerol-3-phosphate by rat liver homogenates and increased incorporation of [1,3-14C] glycerol into hepatic TG by the intact animal [65]. Hepatic TG production changed with a similar time-course characteristic for each diet. However, the 75% fructose diet produced a greater increase in both determinations, reaching a maximum after 11 days. Despite the increase in hepatic TG formation by both high-sugar diets, only the 75% fructose diet resulted in a consistent and sustained increase in serum TG. These results were suggested to be due to differences in the fractional rate of serum TG removal between the two groups. The authors proposed that high glucose intake most likely produces an early acceleration in the fractional rate of TG removal that fully compensates for any increased production, which could be related to increased insulin stimulated-adipose tissue lipoprotein lipase activity [75] and accelerated adipose tissue lipogenesis [76-78]. This is not the case with fructose, which does not stimulate insulin secretion.

Studies dealing with mechanisms underlying fructose-induced lipogenesis provided sufficient evidence in animals [79]. Enzymes implicated in hepatic lipogenesis were found to be increased by high fructose diets: Seven days on 60% fructose diet [80] induced an increase in hepatic sterol regulatory element binding protein (SREBP-1) expression, which is a key transcription factor responsible for regulating fatty acid and cholesterol biosynthesis, as wall as lipogenic gene expression including fatty acid synthase (FAS) and acetyl Co-A carboxylase in mice. It is of interest that glucose feeding could induce, via insulin stimulation, a short-term peak induction of SREB, whereas fructose caused gradual increasing of SREBP-1c activity, providing evidence that lipogenesis can be independent of insulin control, but may depend on carbohydrate availability [81].

Other studies dealing with the effect of high fructose feeding on mitochondrial and peroxisomal β-oxidation found that fructose has been implicated in reducing PPARα in rat hepatocytes. Eight weeks of a high fructose diet induced a decrease in PPARα, which is a ligand activated nuclear hormone receptor responsible for inducing mitochondrial and peroxisomal β-oxidation [82]. Therefore, fructose might induce hepatic cellular lipid accumulation due to decreased lipid oxidation following reducing PPARα.

Of interest, lipid accumulation in fructose-fed rodents has been suggested to be through intestinal flora. Recently, it has been shown the dietary alteration of intestinal flora increased levels of plasma lipopolysaccharides (endotoxin). Fructose fed mice were found to produce endotoxinemia and fatty liver that could be prevented with antibiotic treatment [83], suggesting a bacterial origin of fructose induced fatty liver.

#### Adiposity and fat storage in adipose tissue

Indeed high fructose feeding has been found to cause an increase in adiposity. High dietary fructose intake and increasing body adiposity is clearly linked in both rats submitted to 57% dietary fructose [69,84,85] and in mice consuming fructose containing soft drinks (HFCS, 15%, 61 Kcal/100ml, 52 g/day) [86]. The increased adipose tissue mass in 3 or 6 week-fructose fed rats has been attributed in part to decreased isoproterenol-stimulated lipolysis and to the increased antilipolytic action of insulin [69]. Lipogenesis in rats, however, is found to be shifted to the liver because fructose feeding: 1. activates lipogenic enzymes such as fatty acid synthase and malic enzyme in the liver but not in the adipose tissue [72,87], and 2. depresses conversion of glucose to lipids in adipose tissue [13,87,88]. Nevertheless, a recent study demonstrated that very long periods (6 months) on HFCS might increase adipose tissue fat in Sprague Dawley rats [89].

Similarly, intracellular lipid accumulation in the cytoplasm of muscle fibres has been demonstrated after several weeks of high sucrose diet, not a pure fructose diet, leading to insulin resistance [90].

Therefore, in animals a high fructose diet induces lipogenesis mainly in the liver or muscle fibers but not in the adipose tissue. However the increased adiposity in adipose tissue would most likely be due to decreased lipid mobilization. Various mechanisms were implicated. These results were induced with high doses of fructose either as dietary fructose or as drinks; and therefore, these effects in rodents could no be extrapolated to effects with physiologically significant amounts in humans.

### Acute studies in humans

In an attempt to understand the mechanisms involved in fructose-induced hypertriglyceridemia and its contribution to de novo lipogenesis in an acute setting, in humans, the group of Frayn [91] used a high dose of fructose 0.75g/Kg body weight in a liquid breakfast of mixed macronutrients. [2H2] Palmitate and [U13 C] fructose or [U13 C] glucose were added to trace the handling of dietary fats and the fate of dietary sugars in the body. Compared with glucose, fructose consumed with the fat-containing liquid increased the 4-h appearance of the meal's fatty acid in VLDL. They found, however, that the large amount of fructose used led to impaired triacylglycerol clearance rather than contributing to de novo lipogenesis.

In addition, Parks and co-workers [7] aimed to determine the magnitude by which acute consumption of fructose in a morning bolus would further increase TG concentrations after the next meal. Six healthy subjects consumed carbohydrate boluses of sugar (85g each) in a random order followed by a standard lunch 4 hours later. Subjects consumed either a control test of glucose (100%), a mixture of 50: 50 or 25:75 (wt:wt) glucose:fructose. The investigators demonstrated that post meal lipogenesis increased in proportion to fructose concentration in a beverage: from 7.8% for 100g glucose beverage to 15.9% after a mixture of 50g glucose: 50g fructose and 16.9% after a mixture of 25g glucose: 75g fructose beverage. Body fat synthesis was measured immediately after the sweet drinks were consumed. This study concluded that fructose has an immediate acute lipogenic effect; with greater serum TG level in the morning, and after a subsequent meal, even if consumed as a small amount in a mixture of sugars. The small amount was either 50g or 75g taken with glucose in a beverage. However, it is misleading to suggest that the consumption of a specific food or food ingredient was the cause of obesity and the rise of Type 2 diabetes. Similar results with high fructose-sweetened beverages showed an immediate increase of acute 24-hour TG in obese men and women [92].

On the other hand, the fate of fructose may be its oxidation and not only TG accumulation. Using an oral fructose load of 0.5 or 1 g/Kg (diluted in water), Delarue et al [93] reported that 56% or 59% of fructose load was oxidized over 6-h study. Again, a very high dose of fructose was used to examine this pathway.

The studies cited above used high amounts of fructose with or without labeled fructose to induce hypertriglyceridemia in an acute setting to evaluate underlying mechanisms. We can not draw negative conclusions about moderate amounts of fructose as the cause of obesity epidemic from these studies.

### Chronic studies in humans

**Figure 2:**
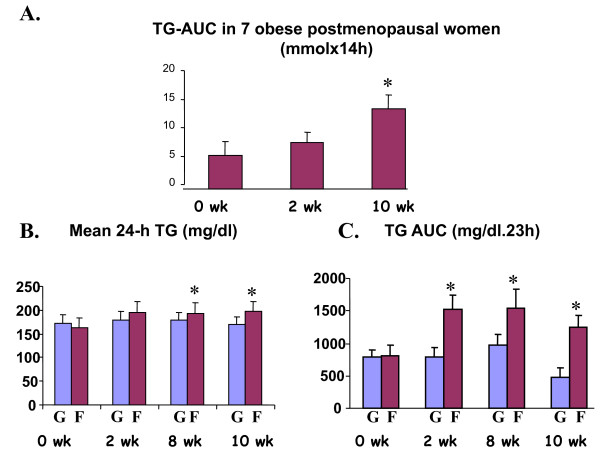
**Postprandial TG responses to fructose- and glucose sweetened beverage consumption. A.** Changes of the area under the curve over 14 h sampling periods before and after 2 and 10 weeks of consuming fructose sweetened beverages at 25% of daily energy in 7 overweight or obese postmenopausal women, values are means ± SE, * :p < 0.05 vs 0wk (*figure adapted from Swarbrick et al (94))*. **B**. Mean 24 hour TG and **C**. TG AUCs (23 h) before and after 2, 8 and 10 week consumption of glucose or fructose-sweetened beverages at 25% of daily energy intake in overweight/obese humans (**G**=glucose group: n= 14; **F= **fructose group: n= 17); values are means ± SEM, * :p < 0.05 vs 0wk in the fructose group ***(figures adapted from Stanhope et al ***[23]***).***

Swarbrick et al [94], evaluated the metabolic effect of 10 week consumption of fructose-sweetened beverages (25% of total carbohydrates). The authors demonstrated that the consumption of fructose-sweetened beverages increased postprandial TG and fasting apo B concentration. They suggested that long-term consumption of diets high in fructose could lead to an increased risk of cardiovascular diseases. Nevertheless, the conclusion was drawn after a study undertaken in only 7 overweight or obese postmenopausal women with special metabolism and a special type of adiposity. Limitations of this study are mainly due to the substantial variations of postprandial TG, (see Figure 2-A). The presented SEMs are great with expected high and overlapping SD values. Moreover, this study in one group consuming fructose sweetened beverages lacks comparison with another group consuming sucrose sweetened beverages.

Later, the same group [23] using a similar protocol, but in a group of overweight/obese subjects (16 men and 16 women), compared the effect of glucose to that of fructose- sweetened beverages providing 25% of energy requirements for 10 weeks in overweight and obese subjects on visceral adiposity, plasma lipids and insulin sensitivity. The carbohydrate intake of these subjects was 25% from sweetened beverages and 30% complex carbohydrates. This means that fructose or glucose represented half of the provided carbohydrates; as mentioned in the study: this amount was higher than 15.8% (the current estimate for the mean intake of total added sugars by Americans [95]). The authors evaluated the effect of the sweetened beverages with an *ad libitum *diet, meaning that subjects could eat as much as they want without any special recommendation or counseling concerning food intake. As expected both groups exhibited significant increase in body weight, fat mass, and waist circumference, without any difference between the two groups. The authors said that visceral adipose volume was significantly increased only in subjects consuming the fructose-sweetened beverages. However, it was not clear how total visceral adipose tissue was measured. The authors cited that they had done a CT scan at the umbilicus level. This means that this was in a one cross section at one level. Moreover, even by DEXA measurements (Dual energy X-ray absorptiometry) visceral or subcutaneous adipose tissue could not be estimated precisely. Therefore, it is misleading to say that in such a study visceral fat is increased by fructose- sweetened beverages. On the other hand, it is not surprising that high amounts of fructose might induce postprandial hypertriglyceridemia as well as increase fasting LDL and apo B. The limitation of this study is the great variations in the SEM presented (figure 2B and 2C). In addition, while it is true that fructose consumption increased the 23-hour postprandial TG AUC as well as the mean 24h TG compared to results before fructose consumption, there was no significant difference between glucose- and fructose-sweetened beverage consumptions (Figure2B and 2C).

Havel et al [92] demonstrated later that the increase in TG excursions during 24 hours (Area under curve) of fructose beverages depends mainly on the degree of insulin resistance of obese subjects.

Recently, Lê et al [96] found that a 7-day hypercaloric high-fructose diet (3.5 g fructose/kg/day, +35% energy intakes) increased ectopic lipid deposition in liver and muscle and fasting VLDL-TG as could be expected with these high amounts. The alteration in plasma lipids was more pronounced in a group of healthy offspring of patients with type 2 diabetes, who might be more susceptible to developing lipid alterations when subjected to high fructose intake. This is in agreement with the finding of the same group in 7 healthy men [22] demonstrating that four weeks of a high fructose diet containing 1.5 g fructose/kg body weight/day increased plasma TG but without causing liver or muscle lipid deposition or insulin resistance in these healthy subjects.

One of the effects of fructose intake is a suppression of plasma free fatty acids, which suggests an inhibition of adipose tissue lipolysis [97]. While this has been confirmed in rats on isolated adipocytes [69], the same effect has been shown in healthy subjects after 7 days on a high fructose diet [98].

In humans, in acute as well as in chronic studies, high (>15% Energy, more than 50g/day), fructose feeding has been found to elevate daylong serum triglycerides in healthy subjects ([17], [99-102]^103^), diabetic patients [104] and overweight/obese subjects [23,105]. Evidence exist that the elevated postprandial triglyceride levels as well as lipid deposition in liver and muscle depend on insulin resistant status of the subjects.

### Epidemiological Studies

**In a longitudinal study **Fung et al [106] found that women who drink two or more servings of sweetened beverages per day may increase their risk of heart disease by 35 per cent. The study evaluated data from 88,520 women 34 to 59 years old participating in the Nurses' Health Study. The women were free of coronary heart disease or diabetes at the end of the study in 1980. Seven food-frequency questionnaires between 1980 and 2002 were used to evaluate dietary habits. While in this study subjects were put on all sweetened beverages. The authors accused fructose, since it had been the major sweetener in the sugar sweetened beverages. However, none of the observational data were able to establish causality.

While most studies have been conducted in adults, rare studies have been done in children.

**Figure 3:**
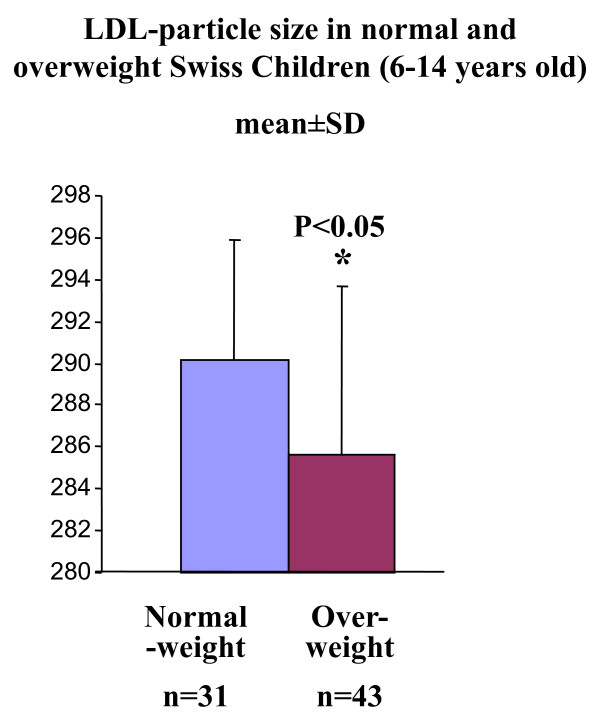
**LDL particle size in 6 to 14 years old Swiss children, values are means ± SD, (*Figure adapted from Aeberli et al ***[4].

Studying normal-weight and overweight 6-14 old Swiss children, Aeberli et al [4] aimed to determine whether LDL particle size is associated with dietary factors and especially with fructose intake. The authors used a **cross-sectional**, and not interventional, study in 74 children and dietary intakes were estimated by using two 24-h recalls and a one-day dietary record. Although there were no significant differences in total fructose intake, the authors concluded that after adjusting the results for adiposity, fructose intake was a significant predictor of LDL particle size, which was significantly smaller in the overweight children than in the normal weight ones. However, upon further examination, these values (Figure 3), the LDL particle size, while described as statistically different, could not have significant clinical impact with only a 1.7% reduction between the two groups with overlapping of values (great SD). This study gave quite a negative image of fructose and reopened the debate on whether fructose consumption itself was a health risk. Again it must be noted that this was a cross sectional study and that the main outcome is based on dietary recalls or dietary records. Dietary recalls, even when validated, can not give precise results, particularly in children, because their ability to record or remember their diet is limited [107,108]. In this study there was no association between fructose consumption and HDL, LDL, total cholesterol or triacylglycerol. The study failed to demonstrate an increase in total fructose intake in the overweight children. However, the authors cited that overweight children consumed significantly less fructose, as a percentage of total fructose, from fruits and vegetables but more fructose, also as a percentage of total fructose, from sweetened drinks and sweets. This is some what misleading, because the absolute amounts of fructose intake from fruits and vegetables or from sweet drinks did not differ significantly between the two groups. In addition, the correlation between LDL size and total fructose intake was poor, β = - 0.245. This poor correlation, however, could not confirm a causal relationship. In a debate entitled "Fructose: Sweet or Bitter for Diabetes" that took place during the 26th Symposium on Diabetes and Nutrition Study Group (DNSG, 2008, Varna, Bulgaria) of the EASD, the author (Dr Isabelle Aeberli) admitted that the problem with fructose is due mainly to the amount utilized and not to fructose itself. Moreover, the generation of small triacylglycerol rich lipoprotein particles, such as generated by fructose, does not itself seem to be a sufficient condition for atherogenesis [109].

### Meta analysis

In a recent meta-analysis Livesey and Taylor [40] examined 60 studies looking at the link between fructose intake on fasting plasma TG and 25 studies dealing with the effect of fructose on postprandial plasma TG in humans. This meta-analysis included different types of subjects: healthy, with impaired fasting glucose, impaired glucose tolerance, type 2 diabetes, subjects with elevated risk of coronary heart disease, and subjects with any form of hyperlipidemia. The authors found that fructose intake < 50 g/d had no significant effect on triacylglycerol post- prandially and ≤ 100g/d had no significant effect on fasting levels but was associated with increased postprandial TG excursions. Consumption of 50 g fructose per day for up to 2 years is without effect on fasting plasma triacylglycerol in healthy individuals [110]. At a daily fructose dose >100g, the effect on fasting triacylglycerol depended on whether sucrose or starch was being exchanged with fructose. This effect was dose dependent, and was lower with increasing the duration of treatment. Different health types and sources of bias were examined showing no significant departure from a general trend.

In another meta-analysis, a Canadian group evaluated the differential effects of isocaloric exchange of fructose for other carbohydrates on triglycerides in peoples with diabetes [111]. They selected 14 papers meeting their criteria out of a total of 725 papers. There was no significant effect of the isocaloic exchange of fructose for CHO on TG with strong heterogeneity. In a further analysis separating patients with type 2 diabetes from those with type 1 diabetes, fructose was found to increase triglycerides in type-2 but not type-1 diabetes. This effect could be detected when high doses of fructose was taken (>65g/d) during short- term (≤4 weeks) and when fructose substituted starch [112,113] but not sucrose [114-116]. Moderate fructose consumption (<50 g/d, or ~10% of metabolizable energy intake) has previously been considered acceptable in diabetics [109,117,118].

Therefore, < 50g/day added fructose by day has no deleterious effect on both fasting and postprandial triglycerides.

## Fructose and insulin resistance

### Evidence from experimental studies in animals

There is much evidence in animal models supporting the notion that fructose when consumed in high amounts contributes to hepatic and peripheral insulin resistance [70,71,119,120]. In rats fed a fructose- rich diet Thorburn et al [120], using the hyperinsulinemic euglycemic clamp method, demonstrated lower insulin stimulated glucose uptake in hindlimb muscles and adipose tissues than in rats fed a dextrose rich diet. A decrease in skeletal and hepatic insulin receptor number, determined by an in situ autoradiography technique, as well as a decrease in their gene expression was found by 66% fructose feeding for 2 weeks in rats [121]. Moreover, decreased insulin-induced insulin receptor phosphorylation was demonstrated in the liver of fructose fed rats[122] A 57% fructose diet induced, similarly, a decrease in insulin stimulated glucose incorporation into lipids but increased the antilipolytic action of insulin in isolated adipocytes of normal rats [13,69].

Three weeks of a 10% fructose-rich diet [123] induced adaptive changes in islets of rats: decreased β-cell mass with increasing apoptotic cells, increased glucose-induced insulin release and islet glucose metabolism, increased glucokinase, but not hexokinase activity. These modifications resulted in an increase of insulin release in spite of marked β-cell mass reduction leading to hyper insulinemia, impaired glucose tolerance and insulin resistance.

Here again, the high fructose fed rats used as a model of insulin resistance to evaluate the islet adaptive changes in such situations (peoples at risk of developing type 2 diabetes). Recently, the group of Havel [124] has demonstrated that 4 months of sustained fructose consumption (20% of energy) accelerate the onset of type 2 diabetes in a model of pylogenic obese type 2 diabetic rats. The presence of an antioxidant with insulin sensitizing activity ameliorates the effect of fructose by improving glucose homeostasis, which is likely due to preserving β-cell function.

Moreover, fructose-fed rats demonstrated a defect in neural insulin signaling pathway in the brain. Decreased insulin stimulated-tyrosine phosphorylation of insulin receptors and insulin receptor substrate 1 (IRS-1) were demonstrated in the fructose-fed hamsters [125]. Also insulin-mediated phosphorylation of residues necessary for activation of another key effector of insulin signalling was markedly decreased.

Nevertheless, high fructose-fed rat model is often used in many studies as a dietary model of insulin resistance [15,126,127]. In rodents, therefore, there is no doubt that high-fructose feeding cause insulin resistance.

### Acute Studies in Humans

In humans, hardly any evidence exists to confirm directly the negative effects of fructose on insulin sensitivity. Fructose has been considered as a therapeutic tool in the diet of diabetic patients due to its low glycemic index [128] and because it's initial metabolic steps do not need insulin [79]. It elicits an increase in energy expenditure that has been suggested to be beneficial for obese subjects with or without diabetes [97,129]. The effect of fructose infusion on hepatic insulin sensitivity in conditions of moderate hyperglycaemia has been studied during hyperglycaemic clamp study with or without infusion of 16.7 μmol/kg/min fructose [130]. The acute fructose infusion induced both extra hepatic and hepatic insulin resistance, which has been suggested to be secondary to an increased intrahepatic glucose 6-phosphate synthesis. These results raise questions as to whether ingested fructose as part of the diet may have the same effects.

### Chronic studies in humans

Consuming an extra 1000 Kcal as fructose, which is a high amount, for one week induced a reduction in both insulin binding and insulin sensitivity when compared to effects after the same amount of glucose in young healthy subjects [131]. In a special case, the presence of fructose as the unique source of carbohydrate in a very low calorie diet (600 Kcal) postponed by two weeks the expected amelioration of a low calorie diet for plasma glucose and insulin levels as well as insulin binding [132].

Moderate fructose intake (1/3 carbohydrate intake), however, in healthy subjects for 2 weeks has no deleterious effect on insulin sensitivity compared to the same amount of sucrose [133,134].

In healthy subjects, consuming up to1.5 g fructose/kg body weight per day for 4 weeks increased plasma triglycerides but without inducing insulin resistance [135]. The authors of the latter study were able, however, to detect early molecular alterations in **only two **skeletal muscle genes. They suggested, therefore, that these alterations could induce later whole body insulin resistance [135]. The same group showed that fructose overfeeding (3.5 g fructose/kg fat-free mass/day, again a high dose) for 6 days produces hepatic insulin resistance in men, whereas these effects are markedly blunted in healthy young men[136].

In diabetic subjects, other chronic studies could not detect any deleterious effects of moderate fructose intakes: 30 g fructose/day compared to starch as a part of 1400 - 1600 Kcal for 8 weeks [112], or one year [137] or 60g fructose/day for 12 weeks, [138] or 6 months [139].

Using high amount of fructose, however, as fructose-sweetened beverages at 25% of energy requirements for 10 weeks, led to an increase in fasting plasma glucose and insulin levels and decreased insulin sensitivity compared to the same amount of glucose sweetened beverages [23].

### Epidemiological studies

In a prospective large cross-sectional study -Nurses Healthy Study I and II- an association was found between high intake of fructose and the high C-peptide concentrations [140]. Due to this association, the authors suggested that fructose intake may play a role in the development of insulin resistance and type 2 diabetes. However, causal relationship could not be identified from this study design.

In a longitudinal study, Janket et al [141] evaluated the relationship between risk of type 2 diabetes and intakes of total caloric sweeteners, sucrose, fructose, glucose and lactose in a cohort of 38,480 female health professionals. Neither fructose, glucose nor sucrose was related to the risk of developing type 2 diabetes. Therefore, no difference could be detected between the different sugars.

While some investigators are able to detect deleterious effects with high doses or could not detect with moderate doses, others found beneficial effects. Koivisto et al [113] demonstrated that the substitution of moderate amounts of fructose (45-65 g/day: 20% of carbohydrate calories) for complex carbohydrates for 4 weeks improves insulin sensitivity in type 2 diabetic patients. Similarly Reiser et al [102] found that patients adapted to 20% of energy as fructose for 5 weeks had improved plasma glucose responses to a glucose charge compared to a group adapted to starch diet. In a group of children with diabetes 1g fructose/kg/day (30g/day maximum) with guar gum for three weeks was found to decrease HBA1c but with increased glucoseuria [142].

In small doses, however, dietary fructose appears to be beneficial in enhancing glucose tolerance [143,144]. The addition of small doses of fructose to a glucose meal can enhance hepatic glucose disposal. Moreover, the addition of small amounts of fructose to orally ingested glucose increases hepatic glycogen synthesis and reduces glycemic responses in subjects with type 2 diabetes [145]. This effect was found to be due to a rise in Fructose-1-P which has an important indirect effect on hepatic glucose metabolism by modulating glucokinase activity which is a key regulatory enzyme required for the formation of glucose -6-P. Glucokinase also is involved in the inhibition of hepatic glucose release by portal hyperglycemia [146]. Fructose-1-P, at low levels antagonizes a glucokinase regulatory protein, enhancing, then, glucokinase activity. Stimulation of hepatic glycogen synthesis by this mechanism may be of potential therapeutic value. However, high doses could be deleterious.

Recently, a meta-analysis [40] demonstrated that fructose intakes from 0 to ≥ 90g/d have a beneficial effect on HbA1c. This meta-analysis was done on a group of studies in healthy, glucose intolerant and type-2 diabetes. The authors, however, are aware that 50 to 100g is a high fructose intake that could affect postprandial triglycerides. Whether a lowering or maintaining of low HbA1c with these doses of fructose would persist is unknown. We could conclude that moderate fructose consumption (<50 g/d, or >10%ME) appears acceptable and potentially beneficial.

## Fructose ingestion acutely elevates blood pressure

Brown and co-workers [147] showed recently that the acute ingestion of both glucose and fructose drinks (60 g) brings about specific hemodynamic responses. Fructose, in particular elicits an increase in blood pressure that could be probably mediated by an increase in cardiac output without compensatory peripheral vasodilatation.

While fructose-induced hypertension is well demonstrated in rodents via various mechanisms [148], in humans long-term demonstration failed. In the Nurses' Health Study, fructose intake was not associated to the risk for developing hypertension [149]. Moreover, recently [136] in a chronic study using high fructose amount of 1.5 g/kg body weight by day for 4 weeks, there was no significant change in mean blood pressure at the end of four week-fructose diets. There is no existing evidence for the relation between fructose and hypertension in humans.

## Fructose consumption and the risk of gout in humans

Prospective data has suggested that consumption of sugar sweetened soft drinks and fructose is strongly associated with an increased risk of gout in men [150]. They concluded that other contributors to fructose intake such as total fruit juice or fructose rich fruits (apples and oranges) were also associated with high risk of gout. In these studies information was provided on intake of soft drinks and fructose through validated food frequency questionnaires. These studies could not confirm a cause and effect relationship. When comparing 5 weeks of fructose consumption to 5 weeks of that of starch (20% of energy), serum uric acid increased with fructose intake [102]. The authors compared a simple sugar to a complex one; therefore, these findings could be simply due to the effect of a refined sugar. This hypothesis is likely, because when comparing 24% of carbohydrates consumed as fructose to that amount consumed as sucrose, no alteration in uric acid level was detected [151]. On the other-hand when a high fructose amount 250-290g/d was taken for 12 days an increase in both plasma and urinary uric acid was found [152]. Others believe that fructose-induced hyperuricaemia occurs mainly in gouty patients [153].

## Fructose and Exercise

Substrate utilization during exercise with glucose and glucose plus fructose ingestion has been an important focus of study. In contrast to glucose during exercise, exogenous fructose has delayed the rate of intestinal absorption [154], lowering the rate of oxidation during exercise [155,156] possibly as a result of its slower absorption rate and the necessity for its conversion to glucose by the liver before oxidation [156]. The combination of fructose and glucose, however, is well absorbed during exercise [157] and may facilitate a higher oxidation than either of the two sugars ingested separately [158]. The ingestion of glucose alone and glucose plus fructose delays exhaustion at 90% peak power by 25 and 40% after 90 minutes of moderate-intensity exercise [159]. While pre-exercise and exercise ingestion of glucose and fructose are of equal values in delaying exhaustion, ingestion of fructose before and during the exercise provide a more constant supply of available glucose to the working muscle [160].

## Other beneficial effects

Dietary fructose (20% of the calories from fructose) enhances mineral balance [161]. Another effect is that the intake of 250 ml of a drink rich in fructose after alcohol consumption will decrease the plasma alcohol levels by 10% [162].

## Conclusions

Certainly high fructose consumption can induce insulin resistance, impaired glucose tolerance, hyperinsulinemia, hypertriglyceridemia, and hypertension in animal models. There is no evidence for similar effects in humans at realistic consumption patterns. Although there are existing data on the metabolic and endocrine effects of dietary fructose that suggest that increased consumption of fructose may be detrimental in terms of body weight and adiposity and the metabolic indexes associated with the insulin resistance syndrome, much more research is needed to fully understand the metabolic effect of dietary fructose in humans. Despite the epidemiological parallel between the marked increase of obesity and fructose consumption, there is no direct evidence linking obesity to the consumption of physiological amounts of fructose in humans (≤ 100g/day). A moderate dose (≤ 50g/day) of added fructose has no deleterious effect on fasting and postprandial triglycerides, glucose control and insulin resistance. There is no existing evidence for a relation between moderate fructose consumption and hypertension. Fructose may induce hyperuricaemia, but mainly in patients with gout.

Beneficial effects of moderate amounts of fructose have also been demonstrated: 1. Fructose seems to decrease appetite when taken in a solution or puddings before a meal, 2. It seems to lower plasma glucose responses to orally ingested glucose via stimulation of hepatic glycogen, when added to the glucose challenge, 3. While pre-exercise and exercise ingestion of glucose and fructose are of equal values in delaying exhaustion, ingestion of fructose before and during the exercise provide a more constant supply of available glucose to the working muscle.

Two new reviews were published during the revision of this manuscript that strengthen our conclusions: The first is an evidence-based review [163] indicating that fructose does not cause biologically relevant changes in TG or body weight when consumed at levels approaching 95th percentile estimates of intake. This review is based on recent guidance developed by the US Food and Drug Administration (FDA) [164]. The second review by Tappy and Lê [37] concluded that: 1) there is no unequivocal evidence that fructose intake at moderate doses is directly related with adverse events in man; 2) there is no direct evidence for more serious metabolic consequences of high fructose corn syrup versus sucrose consumption.

The implications of any balance of effects of fructose on different aspects of metabolism in terms of possible risk to health would need to be ascertained using more direct long-term intervention studies.

## Competing interests

The author declares that they have no competing interests.
